# Transoral endoscopic thyroidectomy via vestibular approach: First case in Saudi Arabia

**DOI:** 10.1016/j.ijscr.2020.04.014

**Published:** 2020-05-07

**Authors:** Hassan M. Al Bisher, Alaa M. Khidr, Badria H. Alkhudair, Fatema S. Alammadi, Arwa H. Ibrahim

**Affiliations:** a Imam Abdulrahman Bin Faisal University, King Fahad University Hospital, Department of Surgery, Saudi Arabia; b Imam Abdulrahman Bin Faisal University, King Fahad University Hospital, Department of Anesthesia, Saudi Arabia; c Imam Abdulrahman Bin Faisal University, Saudi Arabia

**Keywords:** Transoral thyroidectomy, Endoscopic thyroidectomy, Vestibular approach, TOETVA, Scar-less thyroidectomy

## Abstract

**Introduction:**

There has been increased interest in applying the principles of minimally invasive surgery in thyroid surgery which was promoted by Miccoli and his colleagues in 1999. Different surgical techniques since then were introduced, transcervical and extracervical approaches.

**Presentation of case:**

A 33-year-old Saudi female presented with a swelling over the left side of her neck and dysphagia for three years. The patient underwent transoral endoscopic left thyroid lobectomy, isthmusectomy and sampling of the central compartment via vestibular approach (TOETVA). To the best of our knowledge, this is the first case underwent TOETVA successfully in Saudi Arabia and gulf cooperation council countries.

**Discussion:**

TOETVA is a novel promising procedure, an adaptation of the concept of natural orifice transluminal endoscopic surgery (NOTES) with advantages of scar-less result in excellent cosmesis while retaining the advantages of minimally invasive surgery and the potential value of the procedure outside the enhanced cosmesis continuous to be defined.

The cosmetic superiority in avoiding visible scarring must be balanced against operative time, post-operative hospital stays, increased expense, surgical training, and steep learning curve required.

The technique is novel, some thyroid surgeons opted for longer postoperative observation to exclude complications. As experience and confidence in the procedure increase, the length of stay should decrease dramatically.

**Conclusion:**

This procedure is feasible and safe with excellent cosmetic result. It is in its initial stage in Saudi Arabia, gulf cooperation council countries and it has a potential to be performed more frequently in near future.

## Highlights

• Transoral endoscopic thyroidectomy via vestibular approach: First case in Saudi Arabia and gulf cooperation council countries.
•Transoral endoscopic thyroidectomy via vestibular approach is an adaption of the concept of natural orifice transluminal endoscopic surgery (NOTES).
• Transoral endoscopic thyroidectomy via vestibular approach has proven to be feasible and safe procedure.
• Transoral endoscopic thyroidectomy via vestibular approach is truly scar-less with aesthetically pleasing result.

## 1 Introduction

Thyroid nodules are a common clinical problem worldwide, and usually surgical management is the mainstay of their treatment [1].

The conventional, classical transcervical thyroidectomy was pioneered and led by Theodor Kocher in 1898. It leaves unacceptable collar scar to some patients which may cause psychological distress and self-image disturbances in some patients [2,3].

There has been increased interest in applying the principles of minimally invasive surgery which was promoted by Miccoli and his colleagues in 1999. Different surgical techniques since then were introduced, transcervical and extracervical approaches [[4], [5], [6], [7]].

Nowadays, cosmesis plays an important role in choosing the type of surgical technique. It has pushed surgeons to think beyond the limitations of conventional surgery [8].

Transoral endoscopic thyroidectomy via vestibular approach (TOETVA) is the newest techniques, it is truly scar-less thyroidectomy, and provide accessible approach to both thyroid lobes [9,10].

We report the first case of transoral endoscopic thyroidectomy via vestibular approach in Saudi Arabia and gulf cooperation council countries. This work is reported in line with SCARE criteria [11].

## 2 Case report

A 33-year-old Saudi female presented with a swelling over the left side of her neck and dysphagia for three years. Neck examination showed a 4 × 3 cm left thyroid nodule moved with deglutition and it was firm in consistency. Neck ultrasound revealed a solitary left thyroid nodule measure around 3.8 × 2.8 × 1.8 cm. Fine needle aspiration cytology showed features suggestive of benign follicular nodule, Bethesda category II.

In 31st October 2019, the patient underwent transoral endoscopic left thyroid lobectomy, isthmusectomy and sampling of the central compartment via vestibular approach.

Endotracheal intubation was done through the nasal route. Prophylactic preoperative intravenous Cefazolin was given. The oral cavity was cleaned with Betadine prior to incision. Three ports were placed through inferior vestibule of the oral cavity. One midline 10 mm camera port and two lateral 5 mm ports. Sub-platysma plane entered, dissected and created using electro-thermal bipolar vessel sealing device (LigaSure retractable L-Hook™). Operative field was insufflated to a maximum of 6 mm Hg.

**Fig. 1:**
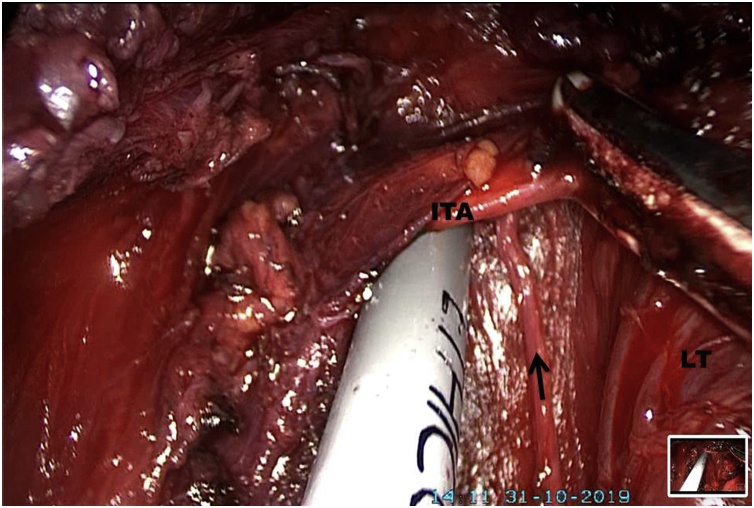
Recurrent laryngeal nerve dissection. Black arrow, left recurrent laryngeal nerve; LT, left thyroid lobe (Retracted); ITA, inferior thyroid artery.

Midline was opened and strap muscle retracted laterally using silk suture at the upper third of the strap muscle. The isthmus of the thyroid gland was identified and divided. The superior and inferior pedicles on the left side were identified and divided. Recurrent laryngeal nerve was identified, dissected and safeguarded (Fig. 1). Intraoperative recurrent laryngeal nerve monitoring was not used. The left lobe and isthmus along with the central sampling was brought out through oral cavity using an endobag. Hemostasis was secured, hemostatic matrix was applied and the vestibular port sites were closed in single layer using a 4−0 chromic catgut suture with an operating room time of 4.5 h. A pressure dressing was applied over the chin and neck for twenty-four hours. Histopathology revealed thyroid adenoma and three reactive lymph nodes.

**Fig. 2:**
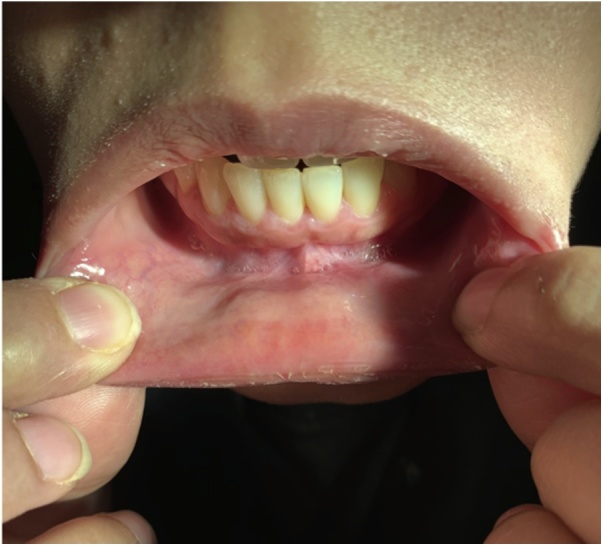
Two months postoperative appearance of the lip following the TOETVA.

**Fig. 3:**
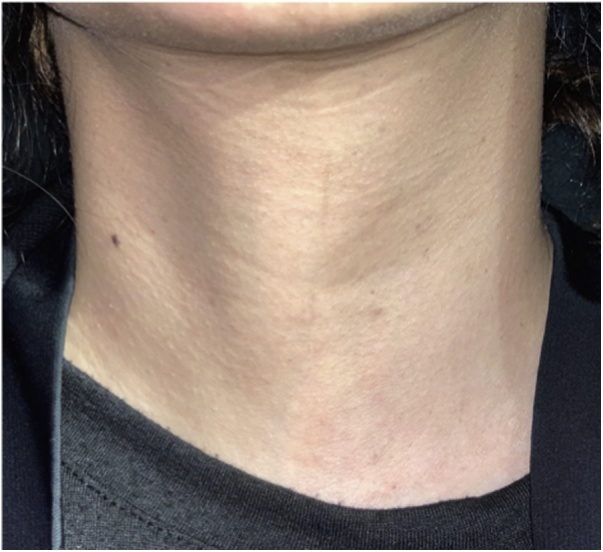
Two months postoperative appearance of the neck following the TOETVA.

Post-operatively, she developed surgical emphysema due to insufflation of the surgical field with carbon dioxide and resolved spontaneously with no intervention and discharged on fourth day. Two months after surgery, the patient was seen in the clinic with no active complaint and thyroid function was within normal limit without hormone replacement therapy; and satisfied with the outcome (Figs. 2,3).

## 3 Discussion

In the mid nineteenth century, thyroid surgery was regarded horrid butchery in America. By the mid twentieth century, it was amongst the most efficient and safest of operations [2].

The gold standard approach for thyroidectomy has been open or conventional surgery; there has been increased interest in applying the principles of minimally invasive to thyroid surgery [9].

The transoral endoscopic technique is one of those approaches; it is an adaptation of the concept of natural orifice transluminal endoscopic surgery to the neck, and it is a technique that promises to improve the aesthetic aspect by offering a scar-less operation while retaining the advantages of minimally invasive surgery [9,10,[12], [13], [14]].

Endoscopic thyroidectomy approaches are yet to be widely employed in clinical practice; however, the drive to improve cosmesis is still important in some patients.

The transoral endoscopic thyroidectomy is a novel promising procedure with advantages of scar-less, natural orifice surgery result in excellent cosmesis and the potential value of the procedure outside the enhanced cosmesis continuous to be defined [10,15].

The cosmetic superiority in avoiding visible scarring must be balanced against operative time, post-operative hospital stays, increased expense, surgical training, and steep learning curve required [16].

Although, it is well known that the transoral endoscopic thyroidectomy technique is challenging and the dissection is difficult, the rate of conversion to conventional thyroidectomy is 1.3 %, which is acceptable [16].

Two different access were reported in this technique. First access, was through floor of the mouth in which the camera port was inserted anterior to the frenulum, whereas the working ports were inserted through the vestibule. The second access, both the camera and working ports were inserted through the vestibule [9,16].

When comparing the complications reported from the two techniques, it was evident that using the floor of the mouth access led to more carbon dioxide embolism, mediastinal emphysema, and both neck and surgical site infection. Otherwise, there was no major difference between the two-reported access [9,14].

Transoral endoscopic thyroidectomy wound is considered as a clean contaminated wound, warranting antibiotic prophylaxis against anaerobic and gram-positive bacteria [17].

Literature review, the patients who underwent transoral endoscopic thyroidectomy spent an average of 4.3 days in the hospital. It could be that given that the technique is novel, some thyroid surgeons opted for longer postoperative observation to exclude complications including bleeding, air way impairment, air embolism and neck space infections. As experience and confidence in the procedure increase, the length of stay should decrease dramatically [9].

Herein, we report a case of left thyroid nodule who underwent transoral endoscopic left thyroid lobectomy, isthmusectomy and sampling of the central compartment via vestibular approach. To the best of our knowledge, this is the first case underwent TOETVA successfully in Saudi Arabia and gulf cooperation council countries.

## 4 Conclusion

The transoral endoscopic thyroidectomy vestibular approach is a new remote access thyroid surgery. This procedure is feasible and safe with excellent cosmetic result. It is in its initial stage in Saudi Arabia, gulf cooperation council countries and it has a potential to be performed more frequently in near future.

## Declaration of Competing Interest

All authors declare that there is no conflict of interests regarding the publication of this paper.

## Sources of funding

No funds or sponsors.

## Ethicalapproval

Case reports are exempted from ethical approval.

## Consent

Written consent was obtained from the patient for publication of this case report and accompanying images. A copy of the written consent is available for review by the Editor-in-Chief of this journal on request.

## Author contribution

Dr. Hassan M. Al Bisher: Main surgeon; conceptualized; writing.

Dr. Alaa M. Khidr: Involved in care of the patient; writing.

Dr. Badria H. Alkhudair: Assistant surgeon; data collection.

Dr. Fatema S. Alammadi: Assistant surgeon; data collection.

Dr. Arwa H. Ibrahim: Assistant surgeon; data collection.

## Registration of research studies

This study didn't require registration.

## Guarantor

Dr. Hassan M. Al Bisher.

## Provenance and peer review

Not commissioned, externally peer-reviewed.
